# Tool use disorders after left brain damage

**DOI:** 10.3389/fpsyg.2014.00473

**Published:** 2014-05-21

**Authors:** Josselin Baumard, François Osiurak, Mathieu Lesourd, Didier Le Gall

**Affiliations:** ^1^Laboratoire de Psychologie des Pays de la Loire, Université d'AngersAngers, France; ^2^Laboratoire d'Etude des Mécanismes Cognitifs, Institut de Psychologie, Université Lyon 2Bron, France; ^3^Unité de Neuropsychologie, Département de Neurologie, Centre Hospitalier Universitaire d'AngersAngers, France

**Keywords:** apraxia, tool use, pantomime, mechanical problem solving, stroke

## Abstract

In this paper we review studies that investigated tool use disorders in left-brain damaged (LBD) patients over the last 30 years. Four tasks are classically used in the field of apraxia: Pantomime of tool use, single tool use, real tool use and mechanical problem solving. Our aim was to address two issues, namely, (1) the role of mechanical knowledge in real tool use and (2) the cognitive mechanisms underlying pantomime of tool use, a task widely employed by clinicians and researchers. To do so, we extracted data from 36 papers and computed the difference between healthy subjects and LBD patients. On the whole, pantomime of tool use is the most difficult task and real tool use is the easiest one. Moreover, associations seem to appear between pantomime of tool use, real tool use and mechanical problem solving. These results suggest that the loss of mechanical knowledge is critical in LBD patients, even if all of those tasks (and particularly pantomime of tool use) might put differential demands on semantic memory and working memory.

## Introduction

Over the past century, a body of evidence has indicated that lesions in the left hemisphere can impair the ability to use tools, hereafter referred to as “apraxia of tool use.” Nevertheless, there is neither consensus on the underlying cognitive processes (semantic knowledge about tool function, sensorimotor knowledge about tool manipulation, mechanical knowledge), nor on the way they are assessed (pantomime of tool use, single tool use, real tool use, mechanical problem solving). So, it may be difficult for students and researchers to obtain a comprehensive overview of tool use impairments after left brain damage. The major aim of this paper is to fill this gap by providing a synthesis of experimental results over the last 30 years. This will lead us to address two crucial issues: (1) The role of mechanical knowledge in tool use, which has received growing attention in recent years; (2) The cognitive processes supporting the most widely employed task, namely, pantomime of tool use.

Before discussing these issues directly, let us specify which studies are eligible for inclusion in the present review. Apraxia covers a wide range of disorders (e.g., constructive apraxia, gait apraxia, apraxia of speech, dressing apraxia) as well as several types of gestures (tool use, symbolic and meaningless gestures). However, we will only emphasize tool use impairment. Besides, the historical ideomotor/ideational apraxia dichotomy has been argued to be confusing, reflecting either a task-based or a process-based distinction (e.g., Hermsdörfer et al., 2006; Osiurak et al., 2011; Lesourd et al., 2013a). Therefore, for the sake of clarity, we decided not to use this dissociation to select studies.

## Tool use assessment

Apraxia of tool use can be assessed in at least four ways depending on the amount of information given to patients.

### Pantomime of tool use

Critical to this task is that patients are asked to demonstrate the use of tools *without holding them* in hand. The input modality may vary (visual presentation of the tool, verbal command, imitation) and the examiner may provide more or less information as to the name of the tool, its function, its usual corresponding object^1^ or the necessity of imagining holding the tool in hand. Imitation tasks can be performed without referring to tool knowledge, as in imitation of meaningless postures (Della Sala et al., 2006; see also Goldenberg, 1995, 1999; Goldenberg and Hagmann, 1997). Therefore, we did not consider results about imitation and we only included studies on pantomime of tool use on visual presentation and/or to verbal command.

### Single tool use

Single tool use consists in demonstrating the use of a tool *while holding it* in hand but *without the usual, corresponding object*. Contrary to pantomimes, the tactile input is present, suggesting that patients do not need to form a mental representation of the tool. Additional information may be provided (name of the tool, the action or the goal of the action) but, for our purpose, we did not take these criteria into account.

### Real tool use

In this task, patients are asked to *actually* use tools *with the usual, corresponding object*. We distinguished between two conditions (no-choice versus choice). In the no-choice condition, patients are presented with only the tool and its corresponding object. In the choice condition, several tools and objects are given. Two criteria can be found in the literature, namely, the presence/absence of tools/objects not useful for the action to be done (i.e., distractors) or the presence/absence of a sequence of at least two actions involving more than two tools/objects (i.e., multiple object task). This latter condition can be viewed as a choice condition since each time an action is performed with two tools/objects (e.g., striking the match on the matchbox), the remaining tools/objects (e.g., the candle) become distractors for this specific action.

### Mechanical problem solving

These tasks require using novel tools in order to solve an unfamiliar tool use situation (e.g., extracting a target from a box or lifting a cylinder). The solution can be found out from the mere observation of the device, perhaps without adopting trial-and-error strategy. This covers situations wherein familiar tools have to be used in a non-conventional way (e.g., screwing a screw with a knife). As for real tool use, two conditions exist: choice (i.e., selection of the correct tools among an array of novel tools) and no-choice (i.e., only the correct, novel tool is present).

## Theoretical background

It is commonly assumed that tool use is supported by two systems: The conceptual and the production system. The role of the conceptual system is to form a mental, tool action representation. Three kinds of knowledge have been proposed in the literature. The first one corresponds to semantic knowledge about tool function, which contains information about the usual relationship between a familiar tool and its corresponding object or the context wherein it can be used (e.g., a hammer is commonly used with a nail and can be found in a workshop; Roy and Square, 1985; Rothi et al., 1991; Buxbaum, 2001). In other words, it refers to allocentric relationships (i.e., tool-object), and is associated with left anterior, temporal lobe lesions (Hodges et al., 2000; Goldenberg and Spatt, 2009; Goldenberg, 2013a).

Second, sensorimotor knowledge about tool manipulation comprises information about the movements associated with the usual manipulation of a specific tool (e.g., the use of a hammer requires ample elbow oscillations; Rothi et al., 1991; Buxbaum, 2001). So, contrary to semantic knowledge, sensorimotor knowledge is supposed to encode egocentric relationships (i.e., tool-user). Damage to the left inferior parietal lobe might impair this kind of knowledge (e.g., Buxbaum and Saffran, 2002; Buxbaum and Kalénine, 2010; Kalénine et al., 2013).

Third, mechanical knowledge provides information about relationships between the physical properties of tools and objects (e.g., hammering requires that the hammer is heavier than the nail; Goldenberg and Hagmann, 1998b; Goldenberg and Spatt, 2009; Osiurak et al., 2010, 2011, 2013; Osiurak, 2014). This kind of knowledge refers to allocentric relationships (i.e., tool-object) and might be also supported by the left inferior parietal lobe (Goldenberg and Spatt, 2009).

The role of the production system is to generate a specific movement pattern by taking into account both the environmental constraints and the tool action representation built by the conceptual system (for discussion, see Osiurak, 2013a,b). The dorsal stream would be the neural basis of this production system (Heilman et al., 1986; Buxbaum, 2001; Binkofski and Buxbaum, 2013).

The aforementioned kinds of knowledge have been suggested to be differentially involved depending on the given task (pantomime of tool use, single tool use, real tool use, mechanical problem solving). Special attention has to be paid to pantomime of tool use given that it might be grounded on processes that are not tool-specific. Indeed, the most widespread interpretation of impaired performance in this task stresses damage to sensorimotor knowledge (i.e., the sensorimotor knowledge hypothesis; Heilman et al., 1982; Buxbaum et al., 2005). However, it has also been hypothesized that it is a non-routine, creative task requiring working memory in order to temporarily maintain information about how the tool has to be held in hand and should be used with the corresponding, absent object (i.e., the working memory hypothesis; Roy and Hall, 1992; Bartolo et al., 2003). At last, pantomime of tool use has been assumed to be nothing else but a kind of symbolic gesture (i.e., the symbolic hypothesis; Goldenberg et al., 2003). In this view, the demonstration by pantomime would aim to communicate the idea of the action rather than to attempt to reproduce the gesture strictly speaking. We shall return to these three hypotheses in more detail below.

## Methods

The purpose of the present paper was to review the experimental data published on pantomime of tool use, single tool use, real tool use and mechanical problem solving since 1985 (i.e., the year Roy and Square published the conception-production model). To this end, several databases (i.e., PubMed, ScienceDirect, Eric, Francis, PBSC, Psycarticles, Web of Knowledge) were searched in 2013–2014 for the following keywords: “tool use,” “object use,” “apraxia,” “limb apraxia,” “ideational apraxia,” “apraxia of tool use,” and “stroke,” “left brain damage,” “left hemisphere.”

### Selection of papers

Only English language experimental studies were included. They had to meet the following criteria:

1. *Presence of right-handed patients with lesions confined to the left hemisphere*. Studies were not included if they involved healthy subjects only or if they investigated disconnection syndromes.
2. *Presence of a control group* consisting of healthy subjects or at least non-neurological patients.
3. *Administration of at least one of the four critical tasks* (i.e., pantomime of tool use, single tool use, real tool use, mechanical problem solving). Pantomime tasks had to be made of “pure” pantomime items, without other types of items such as symbolic gestures (e.g., waving goodbye). Besides, tasks were considered as mechanical problem solving tasks only if patients had to hold a tool to use with an object, and only if it could be achieved through inference rather than trial and error, so as to be comparable with other tool use tasks.
4. *Administration on verbal command, visual presentation or tactile input*. Even though the aforementioned tasks can be administered on imitation, we did not consider this modality because imitation is not supposed to be accounted for by semantic knowledge about tool function, sensorimotor knowledge about tool manipulation or mechanical knowledge (see Roy and Square, 1985; Rothi et al., 1991). Moreover, there is no consistent correlation between production of symbolic gestures on verbal command and on imitation (Heath et al., 2001). Therefore, we focused on verbal, visual and tactile presentation of tools or objects. It is noteworthy that we could have studied modality effects, but we did not do so. Because of methodological heterogeneity in the field of apraxia, this would have led us to generate too many categories with very few studies for each modality, preventing us from drawing firm conclusions.
5. *Availability of quantitative behavioral data* for both patients and controls, allowing us to convert mean performance levels into percentages, and to contrast them. Frequency of impairment among patients, z-scores, number of errors, and kinematic data were not taken into account. Finally, we excluded “redundant” studies (i.e., studies whose data had already been published) for it would have exaggerated some results.

**Figure 1:**
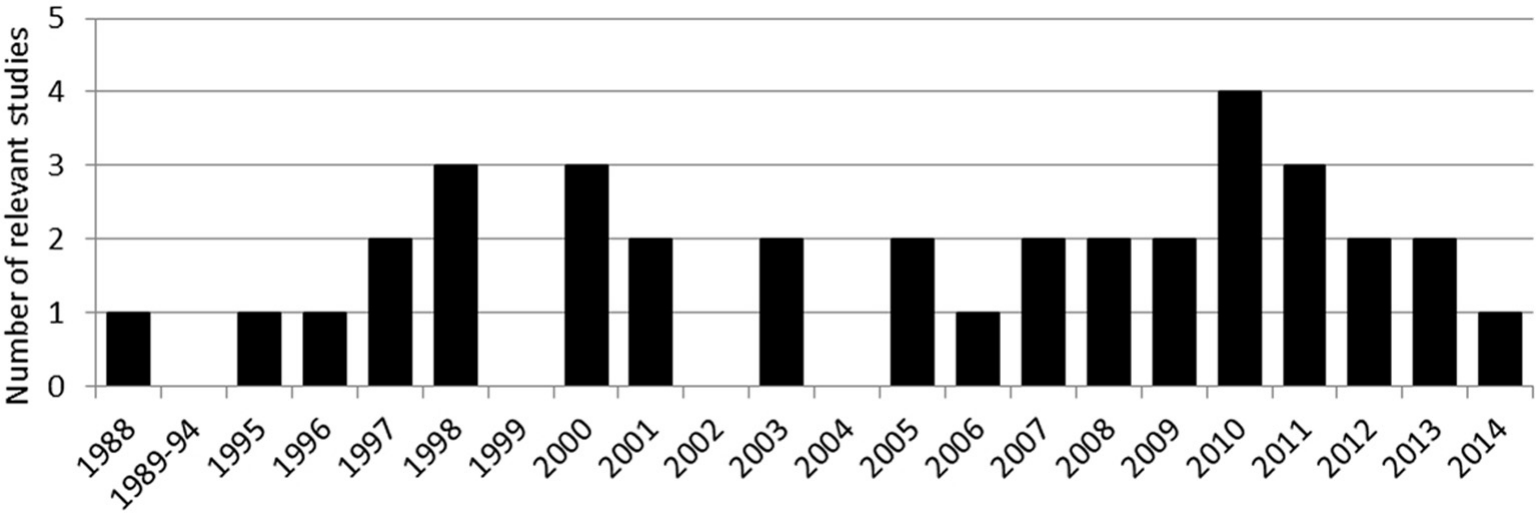
**Repartition of the 36 studies included in the present review over time**.

Our keywords led us to create a corpus of 176 studies. Only 36 out of 176 studies fitted our criteria (see Figure 1). In this pool we counted 59 different tasks, considering that several studies included more than one relevant task. Regarding our criteria, tool use is frequently assessed through pantomime of tool use (25/36, 69%) whereas single tool use (12/36, 33%), real tool use (14/36, 39%) and especially mechanical problem solving (8/36, 22%) were only occasionally investigated over the last 30 years. This can be explained by a lack of consensus in this field (see Dovern et al., 2012).

### Data extraction

In many papers only *apraxic* left-brain damaged (LBD) patients are included, most often on the basis of imitation or pantomime tasks. However, although some manifestations of apraxia are more prevalent following left rather than right hemisphere lesions (Goldenberg, 2009), this is not the case for real tool use and naturalistic actions (Schwartz et al., 1999; Hartmann et al., 2005; Rumiati, 2005). We did not select these studies because our purpose was to analyze the consequences of left-brain-damage, rather than apraxia, on tool use. Indeed, if we did so, this would have led us to follow a pointless, circular reasoning, namely, apraxic patients are apraxic. Nevertheless, we reviewed these studies if they secondarily included non-apraxic LBD patients. In this case, we calculated the mean performance of apraxic and non-apraxic LBD patients (by taking into consideration, of course, the number of patients in each category). We acknowledge that this may be a bias since it does not display the performances of consecutive patients. However we believe it reflects the state of literature, and it prevented us from eliminating too many relevant studies.

### Data analysis

In order to make data from these 36 studies comparable, we converted mean performances and standard deviations into percentages. Then, we calculated the mean performance level for each task, weighted by sample sizes. Furthermore, for each study, we computed the difference between controls' scores minus LBD patients' scores (for a similar method, see Lesourd et al., 2013a,b). This procedure appears suitable for several reasons. First, given the low number of studies available and methodological heterogeneity, it was not relevant to conduct a meta-analysis. Second, the performances of control subjects can vary between studies, therefore focusing on differences rather than raw scores avoids a bias when comparing papers. At last, this procedure expresses the severity of the impairment in each task, which is a good way to determine whether different tasks call upon similar or different cognitive mechanisms (e.g., a difference of 50% in one task and 10% in another may lead us to infer divergent cognitive demands). However, results from this procedure have to be taken with caution in light of the high frequency of ceiling effects in control groups, which can artificially reduce the difference to patients.

## Results

### Comparisons based on weighted means

As can be seen in Table 1, performance of LBD patients is lower for pantomime of tool use than for single and real tool use (mean scores 66, 78, 84%, respectively; range 34–88, 68–100, 72–100), with the two latter producing similar results at first sight. Actually, in terms of level of performance, pantomime of tool use is closer to mechanical problem solving (mean score 68%, range 30–94), than to other conditions.

**Table 1:** **Performances of control subjects and LBD patients (mean scores and standard deviations)**.

	**Patients (*n*)**	**Pantomime of tool use**		**Single tool use**		**Real tool use**		**Mechanical problem solving**	
		**NOR**	**LBD**	**NOR**	**LBD**	**NOR**	**LBD**	**NOR**	**LBD**
Flores-Medina et al., 2014	17	85 (2)	45 (5)	–	–	–	–	–	–
Hermsdörfer et al., 2013	23	–	–	99 (1)	71 (27)	–	–	–	–
Jarry et al., 2013	16	87 (11)	47 (36)	93 (9)	72 (27)	98,8 (3)	76 (29)	92 (10)	58 (33)
Bickerton et al., 2012	74	–	–	–	–	96 (7)	80 (32)	–	–
Hogrefe et al., 2012	24	92 (7)	69 (27)	–	–	–	–	–	–
Poole et al., 2011	30	–	–	–	–	88 (8)	76 (7)	–	–
Papeo et al., 2011	12	–	–	96 (1)	85 (4)	–	–	–	–
Randerath et al., 2011	25	100 (7)	75 (34)	100 (0)	88 (16)	100 (0)	100 (2)	–	–
Randerath et al., 2010	42	–	–	100 (0)	79 (19)	–	–	–	–
Stamenova et al., 2010	42	95 (1)	71 (4)	–	–	–	–	–	–
Vanbellingen et al., 2010	84	88 (12)	58 (32)	–	–	–	–	–	–
Dawson et al., 2010	6	95 (5)	85 (10)	–	–	–	–	–	–
Jacobs et al., 2009	18	–	–	94 (4)	69 (28)	–	–	–	–
Osiurak et al., 2009	20	–	–	–	–	100 (2)	89 (19)	85 (7)	64 (20)
Lunardelli et al., 2008	30	–	–	–	–	–	–	45 (24)	30 (17)
Osiurak et al., 2008	16	93 (6)	71 (30)	–	–	**99 (2)**	**92 (14)**	–	–
Goldenberg et al., 2007^*^	11	93	80	–	–	95	83	100	94
Bartolo et al., 2007	5	92 (4)	44 (33)	–	–	91 (7)	74 (12)	98 (3)	81 (19)
Jax et al., 2006	15	91 (6)	81 (13)	–	–	–	–	–	–
Buxbaum et al., 2005	13	89 (1)	71 (19)	–	–	–	–	–	–
Hartmann et al., 2005	25	93 (1)	66 (5)	–	–	92 (2)	83 (3)	99 (1)	88 (3)
Goldenberg et al., 2003	40	96 (3)	66 (27)	–	–	–	–	–	–
Bartolo et al., 2003	1	97 (5)	60	**100 (0)**	**100**	–	–	–	–
Halsband et al., 2001	13	98	80	–	–	**100**	**98**	–	–
Hanna-Pladdy et al., 2001	14	85	41	–	–	–	–	–	–
Neiman et al., 2000	30	–	–	–	–	98	78	–	–
Cubelli et al., 2000	19	–	–	93	72 (28)	–	–	–	–
Roy et al., 2000	46	93 (3)	87 (8)	–	–	–	–	–	–
Goldenberg and Hagmann, 1998b	42	84	50	–	–	99	92	100	85
Goldenberg and Hagmann, 1998a	35	86 (11)	34 (32)	99 (3)	78 (21)	–	–	–	–
Roy et al., 1998	26	95 (3)	88 (4)	–	–	–	–	–	–
Heilman et al., 1997	21	86 (23)	56 (27)	94 (12)	68 (23)	100 (0)	83 (17)	82 (17)	57 (25)
Schnider et al., 1997	16	98 (2)	78 (21)	100 (0)	93 (10)	–	–	–	–
Belanger and Duffy, 1996	25	91 (5)	71 (14)	90 (3)	77 (12)	–	–	–	–
Foundas et al., 1995	10	–	–	–	–	100 (0)	72 (22)	–	–
Barbieri and De Renzi, 1988	56	97 (4)	76 (23)	–	–	–	–	–	–
Weighted mean		92	66	97	77	97	84	85	68
Minimum mean score		84	34	90	68	88	72	45	30
Maximum mean score		100	88	100	100	100	100	100	94

* *We included this paper although some data have already been published in a larger sample (Hartmann et al., 2005)*.

*Bolded values are non-significant differences*.

### Comparisons based on group differences

**Figure 2:**
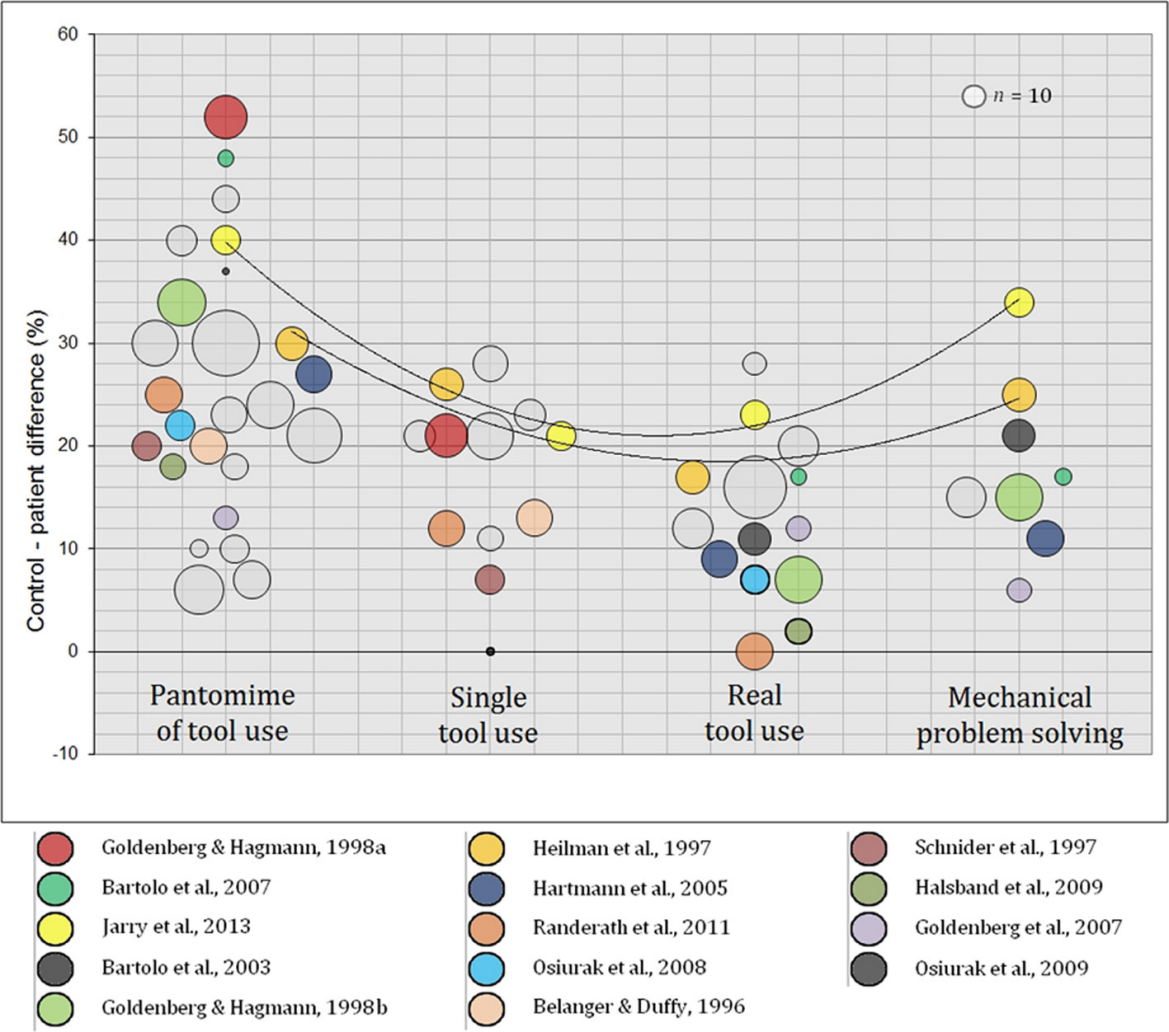
**Differences (in percentage) between control subjects and LBD patients: pantomime of tool use, single tool use, real tool use and mechanical problem solving**. Colored circles correspond to studies that investigated more than one task. Circles in bold are non-significant differences. Curves were drawn for studies that investigated the four tasks.

Control-patient differences are presented in Figure 2 (raw scores are displayed in Table 1). Each circle corresponds to one task in one study. The Y-axis displays the distance in percentage between control subjects and LBD patients: The greater the difference, the higher the impairment. Colors were assigned to some circles in order to stress studies in which two, three or four tasks were administered. Gray circles represent studies that investigated only one of the four tasks. Circles in bold are non-significant differences. Circle surfaces express sample sizes and curves were drawn for the only two studies that investigated all of the four tasks (Heilman et al., 1997; Jarry et al., 2013).

At first sight, there is more discrepancy between studies concerning pantomime of tool use, and there is a gradient from pantomime of tool use (mean difference 25%, range 6–52) to real tool use (mean 13%, range 0–28) with single tool use being intermediary (mean 17%, range 0–28). Actually, the first is systematically more difficult than the two latter if we focus on studies in which at least two tasks were administered (i.e., colored circles in Figure 2).

Second, this gradient is less obvious between single and real tool use. Although previous studies found no significant association between these tasks (Butler, 2002; Bickerton et al., 2012; see also Riddoch et al., 1989), only two studies investigated both of these tasks in respect of our criteria: According to Jarry et al. (2013), both of them are equally difficult whereas Randerath et al. (2011) found real tool use to be much easier. This gap is most likely due to methodological variations since the latter authors employed a very structured task (i.e., patients were assessed with only two items, they did not have to select tools in real tool use and they were provided with verbal information about the action to be done).

Finally mechanical problem solving appears to be the most difficult task after pantomime of tool use (mean difference 18%, range 6–34). More specifically, the control-patient difference is almost always greater than in single and/or real tool use. Only one out of eight studies reported the opposite finding in LBD patients with posterior lesions only (Goldenberg et al., 2007). Nevertheless, real tool use and mechanical problem solving are assessed with a wide array of tasks. In light of these results, it appeared necessary to control for this methodological discrepancy.

### Effect of choice and distractors in real tool use and mechanical problem solving

We divided data from real tool use and mechanical problem solving into two categories: In the no-choice condition, patients are presented with only one tool and its corresponding object (e.g., a match and a matchbox) whereas in the choice condition, they are presented with three or more tools/objects (e.g., a match, a matchbox and a candle; also referred as to multiple object tasks). Within this latter condition, studies were also distinguished by the presence/absence of distractors, defined as tools/objects not useful for the task to be done (e.g., a match, a matchbox and a *hammer*). Situations in which at least two tasks are presented simultaneously (e.g., making coffee, fixing a tape recorder) were judged to include distractors since tools that are useful for one task are useless for the other.

**Figure 3:**
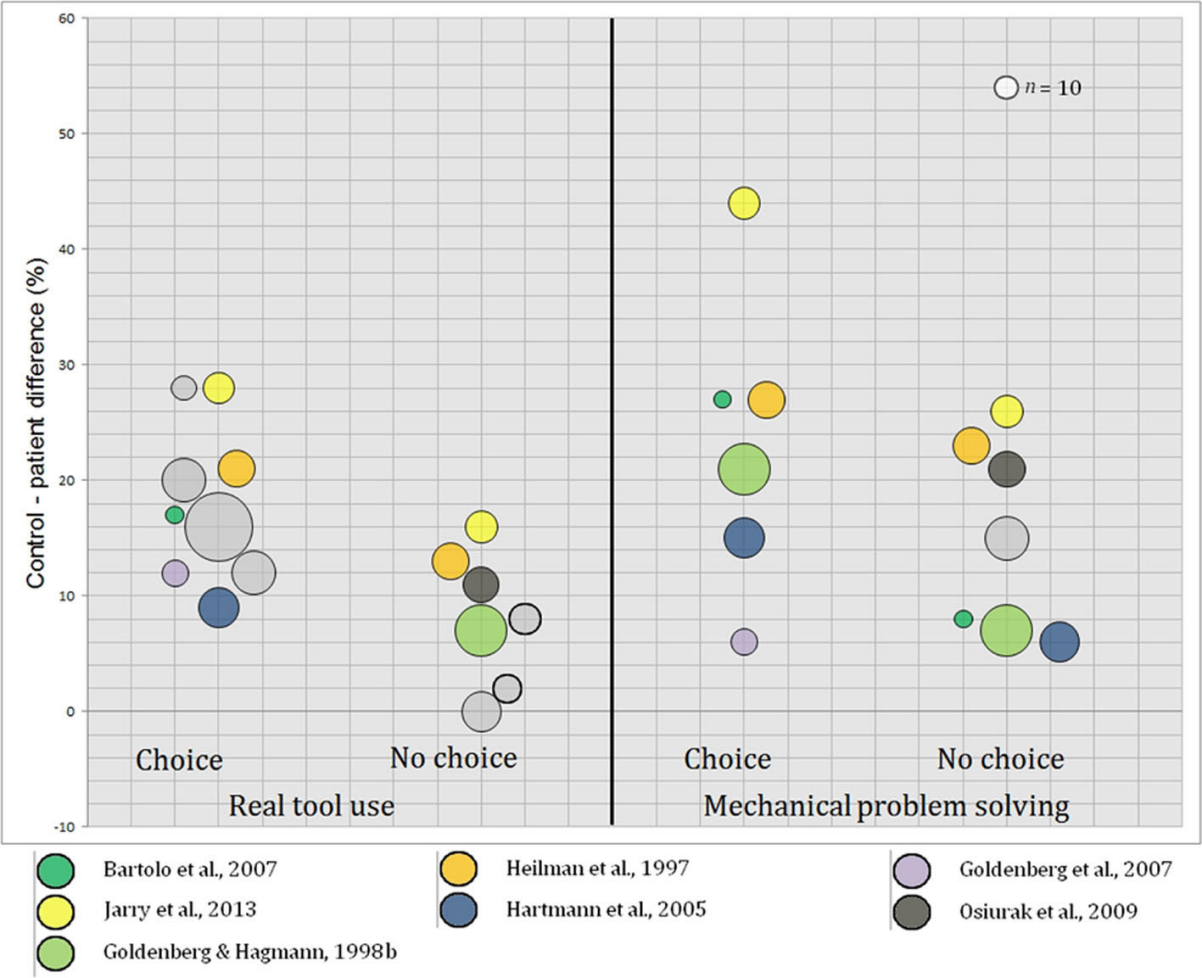
**Differences (in percentages) between control subjects and LBD patients in real tool use (Choice and No-Choice) and mechanical problem solving (Choice and No-Choice)**. Colored circles correspond to studies that investigated more than one condition. Circles in bold are non-significant differences.

As can be seen in Table 2 and Figure 3, only two studies investigated both choice and no choice in both real tool use and mechanical problem solving (Heilman et al., 1997; Jarry et al., 2013). Overall, although mechanical problem solving is more difficult than real tool use, these tasks produce similar results in that reducing the number of tools/objects enhances performances in both conditions. The only study that investigated real tool use (choice) without distractors (Neiman et al., 2000) found similar results.

**Table 2:**
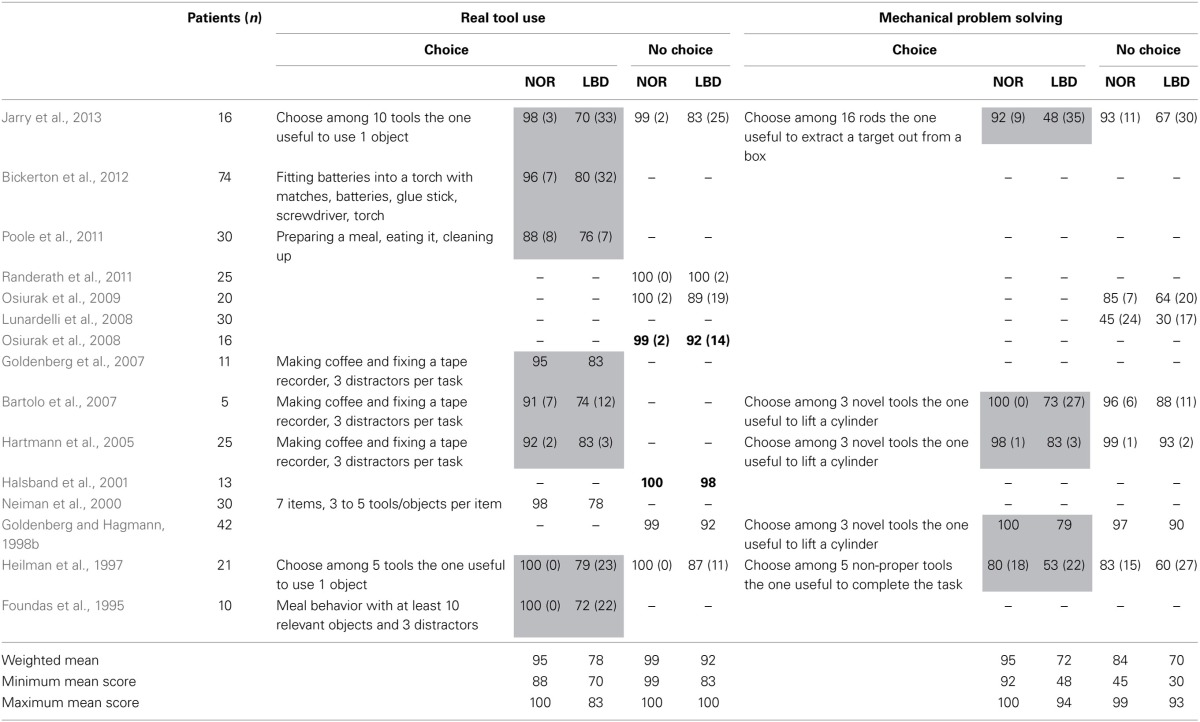
**Effect of choice and distractors in real tool use and mechanical problem solving**.

*Gray scores indicate the presence of distractors (i.e., tools/objects that are not useful for the task to be done)*.

*Bolded values are non-significant differences*.

Finally, as shown in Table 3, pantomime of tool use is more difficult than single tool use, which is more difficult than real tool use (no choice), with choice conditions being intermediary between pantomime of tool use and single tool use. Nevertheless, it is noteworthy that even in no choice condition, LBD patients' performance is significantly impaired as compared to controls.

**Table 3:** **Mean control-patient differences**.

	**Mean control-patient difference (%)**	**Range**
Pantomime of tool use	25	6–52
Single tool use	17	0–28
Mechanical problem solving (no choice)	15	6–26
Real tool use (no choice)	8	0–16
Mechanical problem solving (choice)	23	6–44
Real tool use (choice)	18	9–28

### Associations between tasks

**Figure 4:**
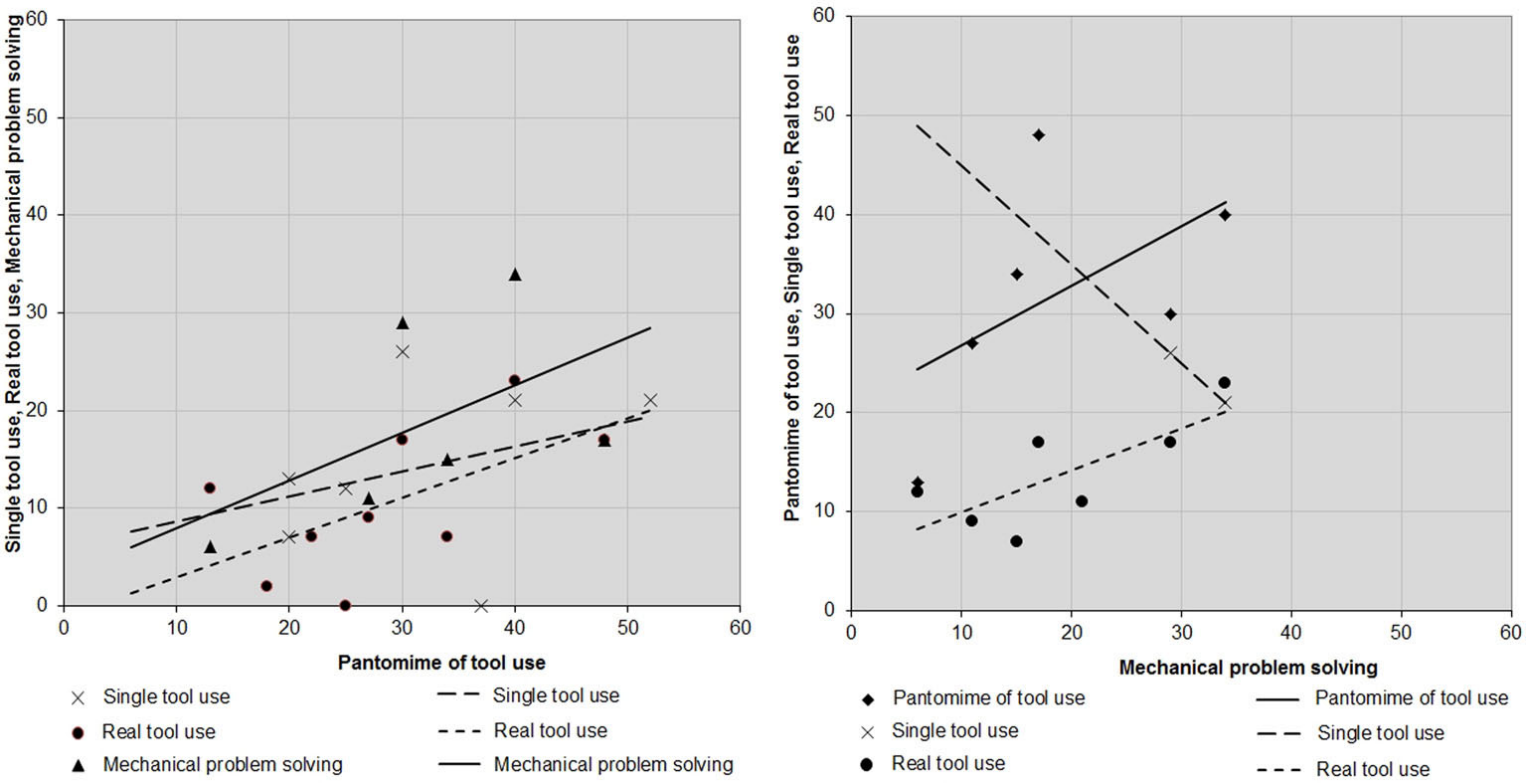
**Associations between pantomime of tool use and single tool use, real tool use, and mechanical problem solving (left panel) and between mechanical problem solving and pantomime of tool use, single tool use and real tool use (right panel)**. Each point corresponds to control-patient differences. Slopes illustrate the degree of association.

We intended to determine whether associations can be found between the tasks of interest. However, given that too few studies explored more than one condition, we only described association tendencies. To this end, we displayed control-patient differences from each study in which at least two tasks where investigated, among pantomime of tool use, single tool use, real tool use and mechanical problem solving (see Figure 4).

As can be seen, stronger positive associations were found between pantomime of tool use, real tool use and mechanical problem solving than between single tool use, real tool use and mechanical problem solving. A negative association was observed between single tool use and mechanical problem solving, but this observation has to be taken with caution given that it concerned only two studies. Interestingly, a slight impairment in mechanical problem solving coincides with more substantial impairment in pantomime of tool use than in real tool use. Furthermore, there is a positive association between mechanical problem solving and pantomime of tool use.

## Discussion

The aim of the present paper was to provide an overview of tool use impairments after left brain damage. More precisely, we shall discuss the role of mechanical knowledge in tool use as well as the cognitive mechanisms supporting pantomime of tool use.

### The role of mechanical knowledge in tool use

Three kinds of conceptual knowledge have been proposed to support real tool use: Semantic knowledge about tool function (Roy and Square, 1985; Rothi et al., 1991; Buxbaum, 2001), sensorimotor knowledge about tool manipulation (Rothi et al., 1991; Buxbaum, 2001) and mechanical knowledge about the physical properties of tools and objects (Goldenberg and Hagmann, 1998b; Goldenberg and Spatt, 2009; Osiurak et al., 2010, 2011). We shall address these hypotheses in turn.

Semantic knowledge provides individuals with information about the usual relationship between familiar tools and objects (e.g., a hammer is usually used with a nail). Therefore, it might be required in at least four situations: When matching pictures of tools with the corresponding, usual object (e.g., hammer/nail) or the context in which they can be used (e.g., hammer/workshop); when it is necessary to select tools/objects to be used together; when pantomiming the use of tools; and when performing single tool use. Indeed, given that objects are not present in the two latter situations, access to semantic knowledge is necessary to produce the right conventional action (e.g., hammering is relevant with a nail but not with a shoe). Interestingly, patients with semantic dementia, who have lost semantic knowledge about tools, have been demonstrated to perform better in no-choice situations and mechanical problem solving, suggesting that these tasks put less demands on functional knowledge (Hodges et al., 2000; Bozeat et al., 2002; Silveri and Ciccarelli, 2009).

In our data, LBD patients perform better in real tool use (no choice; mean control-patient difference 8%, range 0–16) than in pantomime of tool use (25%, 6–52) and single tool use (17%, 0–28). In other words, the more contextual information patients receive, the better they perform. Presumably, this contextual advantage may be a semantic advantage in that the presence of objects in real tool use provides sufficient information and makes retrieval from semantic memory unnecessary. Furthermore, the choice condition of real tool use (18%, 9–28) is more difficult than the no-choice condition of real tool use. These results are consistent with the semantic hypothesis. Nevertheless, patients still perform worse than controls in real tool use (no choice) and mechanical problem solving (18%, 6–34). As a consequence, disruption of semantic knowledge accounts for some, but not all, of tool use impairments. In other words, this kind of knowledge is not sufficient to support tool use (see also Buxbaum et al., 1997).

Sensorimotor knowledge links specific movements to specific tools (e.g., using a hammer requires ample elbow oscillations). Three predictions can be derived from this hypothesis. First, this kind of knowledge should be necessary in any task involving the production of tool-related movements, among which are pantomime of tool use, single tool use and real tool use. Second, choice situations should not be more difficult than no-choice situations because the same movement is required in both cases (e.g., hammering does not vary depending on the number of tools on the desk). Third, the loss of sensorimotor knowledge should not interfere in the use of novel tools, such as in mechanical problem solving.

Our results do not confirm these predictions. Indeed, LBD patients are not impaired to a similar extent in pantomime of tool use (25%), single tool use (17%), and real tool use (no-choice, 8%). Moreover, the choice condition of real tool use is more difficult than the no-choice condition of real tool use even though this dissociation has been assessed in only two studies (Heilman et al., 1997; Jarry et al., 2013) and remains to be confirmed. At last, the sensorimotor hypothesis does not account for impaired performance of LBD patients in mechanical problem solving. On these accounts, experimental data did not prove that apraxia of tool use in LBD patients is due to the loss of sensorimotor knowledge.

Finally, mechanical knowledge about the physical properties of tools and objects (e.g., hammering requires that the hammer is heavier than the nail) may be necessary to use both familiar and novel tools, and might be supported by the left inferior parietal lobe (Goldenberg and Spatt, 2009). So, LBD patients are supposed to be concurrently impaired in both of these tasks.

Overall, our results confirmed this prediction (real tool use, mean control-patient difference 13%; mechanical problem solving: 18%). Moreover, LBD patients are constantly impaired in mechanical problem solving and, in studies that investigated both conditions, failure to solve mechanical problems was systematically associated with failure to use familiar tools (Heilman et al., 1997; Goldenberg and Hagmann, 1998b; Hartmann et al., 2005; Bartolo et al., 2007; Goldenberg et al., 2007; Osiurak et al., 2009; Jarry et al., 2013). Additionally, as shown in Figure 4, there is a clear positive association between the two tasks. These results lead us to suggest that mechanical knowledge is necessary to use familiar tools and objects.

To conclude, experimental data obtained over the last 30 years indicate that real tool use might be supported by at least two kinds of knowledge, both of them referring to allocentric relationships: Semantic knowledge about tool function and mechanical knowledge. These two types of knowledge might partially compensate for each other, in that studies on semantic dementia already described impaired use of familiar tools in the context of preserved mechanical problem solving (Hodges et al., 2000; Bozeat et al., 2002; Silveri and Ciccarelli, 2009). However, to our knowledge, this pattern has never been found in LBD patients. On the other hand, the reverse pattern (i.e., impaired, but better performance in real tool use than in mechanical problem solving; see Table 1) was frequently observed, suggesting that loss of mechanical knowledge can be partially compensated by intact semantic knowledge although it is critical to account for tool use disorders in LBD patients. We shall now discuss the cognitive processes underlying pantomime of tool use.

### The cognitive processes supporting pantomime of tool use

Three hypotheses have been proposed to explain the cognitive basis of pantomime of tool use: The sensorimotor knowledge hypothesis (Heilman et al., 1982; Buxbaum et al., 2005), the symbolic hypothesis (Goldenberg et al., 2003) and the working memory hypothesis (Roy and Hall, 1992; Bartolo et al., 2003).

According to the sensorimotor knowledge hypothesis, pantomime of tool use requires individuals to implicitly recover gesture representations that contain invariant, egocentric relationships, and that are specific to particular tools. Therefore, as suggested above, there should be no difference between pantomime of tool use, single tool use and real tool use. Indeed, because these representations are egocentric and invariant, the presence/absence of tools and objects should not modify control-patient differences. However, the present review confirmed that pantomime of tool use is much more difficult than real tool use (see also Riddoch et al., 1989; Roy and Hall, 1992; Butler, 2002; Bartolo et al., 2003; Bickerton et al., 2012). Moreover, pantomime of tool use seems to be poorly associated with single tool use compared with real tool use and even mechanical problem solving (see Figure 4). These results thus do not favor the sensorimotor knowledge hypothesis.

The symbolic hypothesis assumes that defective pantomime of tool use is due to asymbolia, that is, a “general inability to express concepts by means of learned signs” (Goldenberg et al., 2003). As an example, drawing from memory implies to select typical features of the object to be drawn (e.g., the shape of both the handle and head of a hammer). Presumably, asymbolia should impair any activity that requires access to semantic memory, such as language, drawing from memory and pantomime of tool use (see Goldenberg, 2013b). Indeed, this hypothesis also presumes that pantomimes are part of communicative gestures in that they require patients to select distinctive features of the sensory appearance of absent tools/objects (e.g., the shape of the handle of a hammer) and to abstract properties that do not contribute to recognizability (e.g., the color or the material of the handle) in order to produce a canonical, recognizable gesture.

So, pantomime of tool use should be more difficult than single tool use since in the latter, patients do not need to communicate the idea of the tool because they already handle it. Our data are consistent with this hypothesis: Pantomime of tool use (mean control-patient difference 25%) and single tool use (17%) appear to be weakly associated and the first is consistently more difficult than the latter over studies. Nevertheless, pantomime of tool use is closer to mechanical problem solving than to single tool use (see Table 3, Figures 3, 4) and in previous studies, asymbolia alone could not account for pantomime disturbances in LBD patients (Goldenberg et al., 2003). Further research is thus required on this point.

In line with the working memory hypothesis, pantomiming the use of tools leads individuals to form a mental representation of the tool in hand, the object on the desk and the action to be performed. Once this layout has been imagined, it has to be maintained in working memory until the gesture is finished. This implies that holding a tool in hand and/or seeing the object provides cues, hence reducing the degrees of freedom and so the number of possible errors (Roy and Hall, 1992; Bartolo et al., 2003). As a consequence, the presence of actual tools reduces the load on working memory and enhances performance. The gradient we already described is consistent with this hypothesis.

To sum up, the present review found the working memory hypothesis and, to a lesser extent, the symbolic hypothesis, to be most relevant as regards pantomime of tool use. On the other hand, the sensorimotor knowledge hypothesis remains to be demonstrated. Another key finding is the similar difficulty level and the relationship between mechanical problem solving and pantomime of tool use (see Table 3 and Figure 4). Previous studies reported significant correlations between these tasks (Heilman et al., 1997; Goldenberg and Hagmann, 1998b; Jarry et al., 2013). This finding is not compatible with cognitive models of apraxia (Roy and Square, 1985; Rothi et al., 1991; Buxbaum, 2001) but rather suggests that pantomime of tool use is a composite task that may call for mechanical knowledge, in addition with semantic knowledge and working memory. In fact, this task can be viewed as a kind of problem solving for it may require forming a mental representation through identification and combination of distinctive features of tools and actions (see Goldenberg et al., 2003; Goldenberg, 2009) or, put differently, technical means and technical ends (Osiurak et al., 2010, 2011).

Before concluding, let us discuss results indicating differences between choice and no choice conditions. On the whole, the presence of numerous tools seems to be a major obstacle to LBD patients but not to control subjects. Note that although this finding is intuitive, the cognitive models of apraxia (Roy and Square, 1985; Rothi et al., 1991; Buxbaum, 2001) do not address the issue of how humans choose tools and objects. Interestingly, the choice effect is true for familiar as well as novel tools and, as a result, questions the relationship between mechanical knowledge and tool substitutions. Unfortunately, only one of the selected studies investigated real tool use (choice) without distractors (Neiman et al., 2000). Consequently, it remains unknown whether LBD patients fail multiple object tasks because of a planning impairment, interference from distractors or inability to select and combine useful/useless tools. Nevertheless, these results remain to be confirmed because ceiling effects prevented us from computing the real difference between choice and no-choice conditions in control group. Therefore, such a difference among patients could be accounted for by the intrinsic difficulty of choice conditions. Future research is needed to disentangle the origin of the choice effect in LBD patients.

## Conclusion

To conclude, pantomime of tool use, single tool use, real tool use and mechanical problem solving seem to have at least one cognitive mechanism in common, which may be the ability to retrieve mechanical knowledge on the basis of identification and combination of distinctive features of tools and objects. Nevertheless, each task calls for differential demands depending on presence/absence, familiarity/novelty and number of tools/objects (see Table 4). This theoretical distribution challenges the idea that tool use in general, and pantomime of tool use in particular call for sensorimotor knowledge. Note also that data reported here focus on left brain damage but do not exclude a role of the right hemisphere in tool use (Schwartz et al., 1999; Hartmann et al., 2005; Rumiati, 2005). In sum, although apraxia of tool use is classically viewed as a disorder of movement representations/motor control, the present review emphasizes that apraxia of tool use in LBD patients may be first and foremost a cognitive disorder involving the understanding of how tools and objects have to be used together (Osiurak et al., 2010, 2011; Goldenberg, 2013a).

**Table 4:** **Cognitive demands depending on the task**.

	**Pantomime of tool use**	**Single tool use**	**Real tool use**	**Mechanical problem solving**
Semantic knowledge about tool function and context	+	+	+	−
Mechanical knowledge about physical properties of tools/objects	+	+	+	++
Working memory	++	+	−	−
Production system	+	+	+	+

*^++^High demands on the cognitive process*.

*^+^Moderate demands*.

*^−^Low or absent demands*.

### Conflict of interest statement

The authors declare that the research was conducted in the absence of any commercial or financial relationships that could be construed as a potential conflict of interest.

## Footnotes

^1^We shall use the terms tool and object to refer to the implement performing the action (e.g., screwdriver) and the recipient of the action (e.g., screw), respectively.
