# Supplementary material for: Tool use disorders after left brain damage
Source: Front Psychol. 2014 May 21;5:473. doi: 10.3389/fpsyg.2014.00473 (PMC4033127; doi:10.3389/fpsyg.2014.00473)
Supplement: Supplementary file 1 [file DataSheet1.DOCX]

Supplementary table 1. Comparison between two methods to compute control-patients differences in pantomime of tool use, single tool use, real tool use and mechanical problem solving.

|  | Method 1  CSscore - LBDscore | | | | Method 2  100-((LBDscore/CSscore)*100) | | | |
| --- | --- | --- | --- | --- | --- | --- | --- | --- |
|  | PTU | STU | RTU | MPS | PTU | STU | RTU | MPS |
| Flores-Medina et al. (2014) | 40 |  |  |  | 47 |  |  |  |
| Hermsdörfer et al. (2013) |  | 28 |  |  |  | 28 |  |  |
| Jarry et al. (2013) | 40 | 21 | 23 | 34 | 46 | 23 | 23 | 37 |
| Bickerton et al. (2012) |  |  | 16 |  |  |  | 17 |  |
| Hogrefe et al. (2012) | 23 |  |  |  | 25 |  |  |  |
| Poole et al. (2011) |  |  | 12 |  |  |  | 14 |  |
| Papeo et al. (2011) |  | 11 |  |  |  | 11 |  |  |
| Randerath et al. (2011) | 25 | 12 | 0 |  | 25 | 12 | 0 |  |
| Randerath et al. (2010) |  | 21 |  |  |  | 21 |  |  |
| Stamenova et al. (2010) | 24 |  |  |  | 25 |  |  |  |
| Vanbellingen et al. (2010) | 30 |  |  |  | 34 |  |  |  |
| Dawson et al. (2010) | 10 |  |  |  | 11 |  |  |  |
| Jacobs et al. (2009) |  | 23 |  |  |  | 27 |  |  |
| Osiurak et al. (2009) |  |  | 11 | 21 |  |  | 11 | 25 |
| Lunardelli et al. (2008) |  |  |  | 15 |  |  |  | 33 |
| Osiurak et al. (2008) | 22 |  | 7 |  | 24 |  | 7 |  |
| Goldenberg et al. (2007) * | 13 |  | 12 | 6 | 14 |  | 13 | 6 |
| Bartolo et al. (2007) | 48 |  | 17 | 17 | 52 |  | 19 | 17 |
| Jax et al. (2006) | 10 |  |  |  | 11 |  |  |  |
| Buxbaum et al. (2005) | 18 |  |  |  | 20 |  |  |  |
| Hartmann et al. (2005) | 27 |  | 9 | 11 | 29 |  | 10 | 11 |
| Goldenberg et al. (2003) | 30 |  |  |  | 31 |  |  |  |
| Bartolo et al. (2003) | 37 | 0 |  |  | 38 | 0 |  |  |
| Halsband et al. (2001) | 18 |  | 2 |  | 18 |  | 2 |  |
| Hanna-Pladdy et al. (2001) | 44 |  |  |  | 52 |  |  |  |
| Neiman et al. (2000) |  |  | 20 |  |  |  | 20 |  |
| Cubelli et al. (2000) |  | 21 |  |  |  | 23 |  |  |
| Roy et al. (2000) | 6 |  |  |  | 6 |  |  |  |
| Goldenberg & Hagmann (1998b) | 34 |  | 7 | 15 | 40 |  | 7 | 15 |
| Goldenberg & Hagmann (1998a) | 52 | 21 |  |  | 60 | 21 |  |  |
| Roy et al. (1998) | 7 |  |  |  | 7 |  |  |  |
| Heilman et al. (1997) | 30 | 26 | 17 | 25 | 35 | 28 | 17 | 30 |
| Schnider et al. (1997) | 20 | 7 |  |  | 20 | 7 |  |  |
| Belanger & Duffy (1996) | 20 | 13 |  |  | 22 | 14 |  |  |
| Foundas et al. (1995) |  |  | 28 |  |  |  | 28 |  |
| Barbieri & De Renzi (1988) | 21 |  |  |  | 22 |  |  |  |
| Mean | 26 | 17 | 13 | 18 | 29 | 18 | 13 | 22 |
| Minimum difference | 6 | 0 | 0 | 6 | 6 | 0 | 0 | 6 |
| Maximum difference | 52 | 28 | 28 | 34 | 60 | 28 | 28 | 37 |

PTU: Pantomime of tool use; STU: Single tool use; RTU: Real tool use; MPS: Mechanical problem solving; CS: Control subjects; LBD: Left brain-damaged.
