# Supplementary material for: Tool use disorders after left brain damage
Source: Front Psychol. 2014 May 21;5:473. doi: 10.3389/fpsyg.2014.00473 (PMC4033127; doi:10.3389/fpsyg.2014.00473)
Supplement: Supplementary file 2 [file DataSheet2.DOCX]

Supplementary table 2. Comparison between two methods to compute control-patients differences in real tool use (choice and no-choice) and mechanical problem solving (choice and no-choice).

|  | Method 1  CSscore - LBDscore | | | | Method 2  100-((LBDscore/CSscore)*100) | | | |
| --- | --- | --- | --- | --- | --- | --- | --- | --- |
|  | RTU  Choice | RTU  No choice | MPS  Choice | MPS  No choice | RTU  Choice | RTU  No choice | MPS  Choice | MPS  No choice |
| Jarry et al. (2013) | 28 | 16 | 44 | 26 | 29 | 16 | 48 | 28 |
| Bickerton et al. (2012) | 16 |  |  |  | 17 |  |  |  |
| Poole et al. (2011) | 12 |  |  |  | 14 |  |  |  |
| Randerath et al. (2011) |  | 0 |  |  |  | 0 |  |  |
| Osiurak et al. (2009) |  | 11 |  | 21 |  | 11 |  | 25 |
| Lunardelli et al. (2008) |  |  |  | 15 |  |  |  | 33 |
| Osiurak et al. (2008) |  | 8 |  |  |  | 8 |  |  |
| Goldenberg et al. (2007) | 12 |  | 6 |  | 13 |  | 6 |  |
| Bartolo et al. (2007) | 17 |  | 27 | 8 | 19 |  | 27 | 8 |
| Hartmann et al. (2005) | 9 |  | 15 | 6 | 10 |  | 15 | 6 |
| Halsband et al. (2001) |  | 2 |  |  |  | 2 |  |  |
| Neiman et al. (2000) | 20 |  |  |  | 20 |  |  |  |
| Goldenberg & Hagmann (1998) |  | 7 | 21 | 7 |  | 7 | 21 | 7 |
| Heilman et al. (1997) | 21 | 13 | 27 | 23 | 21 | 13 | 34 | 28 |
| Foundas et al. (1995) | 28 |  |  |  | 28 |  |  |  |
| Mean | 18 | 8 | 23 | 15 | 19 | 8 | 25 | 19 |
| Minimum difference | 9 | 6 | 13 | 8 | 7 | 6 | 15 | 12 |
| Maximum difference | 28 | 16 | 44 | 26 | 29 | 16 | 48 | 33 |

PTU: Pantomime of tool use; STU: Single tool use; RTU: Real tool use; MPS: Mechanical problem solving; CS: Control subjects; LBD: Left brain-damaged.
